# CD34 Identifies a Subset of Proliferating Microglial Cells Associated with Degenerating Motor Neurons in ALS

**DOI:** 10.3390/ijms20163880

**Published:** 2019-08-09

**Authors:** Mariángeles Kovacs, Emiliano Trias, Valentina Varela, Sofia Ibarburu, Joseph S. Beckman, Ivan C. Moura, Olivier Hermine, Peter H. King, Ying Si, Yuri Kwon, Luis Barbeito

**Affiliations:** 1 Institut Pasteur de Montevideo, Montevideo 11400, Uruguay; 2 Linus Pauling Institute, Department of Biochemistry and Biophysics, Environmental Health Sciences Center, Oregon State University, Corvallis, OR 97331, USA; 3 Imagine Institute, Hôpital Necker, 75015 Paris, France; 4 INSERM UMR 1163, Laboratory of Cellular and Molecular Mechanisms of Hematological Disorders and Therapeutic Implications, 75015 Paris, France; 5 Paris Descartes–Sorbonne Paris Cité University, Imagine Institute, 75006 Paris, France; 6 CNRS ERL 8254, INSERM U1163, Université Paris Descartes, 75006 Paris, France; 7 Laboratory of Excellence GR-Ex, 75015 Paris, France; 8 Equipe Labélisée par la Ligue Nationale contre le cancer, 75013 Paris, France; 9 AB Science, 75008 Paris, France; 10 Department of Hematology, Hôpital Necker, 75015 Paris, France; 11 Centre national de référence des mastocytoses (CEREMAST), 75743 Paris, France; 12 Department of Neurology, University of Alabama at Birmingham, Birmingham, AL 35294, USA; 13 Birmingham Veterans Affairs Medical Center, Birmingham, AL 35294, USA

**Keywords:** microglia, CD34, amyotrophic lateral sclerosis, misfolded SOD1, motor neurons

## Abstract

Amyotrophic lateral sclerosis (ALS) is characterized by degeneration of upper and lower motor neurons accompanied by proliferation of reactive microglia in affected regions. However, it is unknown whether the hematopoietic marker CD34 can identify a subpopulation of proliferating microglial cells in the ALS degenerating spinal cord. Immunohistochemistry for CD34 and microglia markers was performed in lumbar spinal cords of ALS rats bearing the SOD1^G93A^ mutation and autopsied ALS and control human subjects. Characterization of CD34-positive cells was also performed in primary cell cultures of the rat spinal cords. CD34 was expressed in a large number of cells that closely interacted with degenerating lumbar spinal cord motor neurons in symptomatic SOD1^G93A^ rats, but not in controls. Most CD34^+^ cells co-expressed the myeloid marker CD11b, while only a subpopulation was stained for Iba1 or CD68. Notably, CD34^+^ cells actively proliferated and formed clusters adjacent to damaged motor neurons bearing misfolded SOD1. CD34^+^ cells were identified in the proximity of motor neurons in autopsied spinal cord from sporadic ALS subjects but not in controls. Cell culture of symptomatic SOD1^G93A^ rat spinal cords yielded a large number of CD34^+^ cells exclusively in the non-adherent phase, which generated microglia after successive passaging. A yet unrecognized CD34^+^ cells, expressing or not the microglial marker Iba1, proliferate and accumulate adjacent to degenerating spinal motor neurons, representing an intriguing cell target for approaching ALS pathogenesis and therapeutics.

## 1. Introduction

Neuroinflammation is a pathological hallmark of amyotrophic lateral sclerosis (ALS), causally associated with the progressive degeneration of upper and lower motor neurons [1,2]. At sites of motor neuron and axonal damage, reactive glial and infiltrating immune cells orchestrate a characteristic inflammatory microenvironment [3,4]. In particular, microglia display active proliferation and profound phenotypic changes in ALS subjects and ALS animal models [5,6]. A large body of evidence indicates that microglia play a crucial pathogenic role in accelerating motor neuron degeneration [7,8]. However, the complete understanding of different microglia cell phenotypes in ALS pathogenesis remains elusive.

In particular, the ALS rat model expressing the SOD1^G93A^ mutation shows extensive microglia pathology concurrent with paralysis onset and progression [9,10,11], with extensive proliferation and development of aberrant or senescent phenotypes and formation of multinucleated giant cells [10,12]. Activated microglia bearing SOD1 mutations in rodents also exert toxicity to motor neurons [7] and contribute to accelerated progression of motor neuron disease in a non-cell autonomous manner [13]. In SOD1^G93A^ rats, microglia can transform into a distinct population of cells exhibiting an aberrant phenotype [9] and a potent neurotoxic effect on cultured motor neurons [14,15]. Notably, aberrant microglia are localized in the vicinity of spinal motor neurons after paralysis onset. Downregulation of aberrant glia through pharmacological inhibition of the receptor CSF-1R results in an extension of post-paralysis survival in rat and mouse models of ALS [16,17], suggesting their potential as drug targets to halt or slow disease progression. Given these facts, we aimed to identify different and as yet unknown microglia-related phenotypes that may also emerge and interact with degenerating motor neurons during disease progression in ALS.

CD34 is a transmembrane highly glycosylated protein, which has been extensively used as a marker of hematopoietic stem cells [18] and non-hematopoietic progenitor cells [19,20]. CD34 can regulate trafficking and migration of hematopoietic progenitor cells [20], which eventually may migrate to the CNS and differentiate into microglia [21]. Following CNS damage, CD34-expressing microglia have been found in affected regions [21,22,23], which are also characterized by microgliosis and blood–brain barrier damage. Because the identification of CD34^+^ cells in ALS remains elusive, we reasoned that this marker may allow the identification of a proliferating subset of microglia in the spinal cord of SOD1^G93A^ rats and autopsied ALS patients.

SOD1^G93A^ rats are characterized by the development of an adult-onset rapid progressing paralysis accompanied by dramatic microgliosis [9,10,11]. We took advantage of this particular SOD1^G93A^ model to analyze CD34 expression along the course of paralysis progression. Here, we report a massive increase of cells expressing CD34 after paralysis onset, which proliferate and co-express myeloid markers. Notably, numerous CD34^+^ cells were associated with motor neurons in sporadic ALS and SOD1^G93A^ rat spinal cords. In cell culture, CD34^+^ cells yielded microglia upon successive passages, suggesting that this marker identifies a population of proliferating microglia involved in ALS pathogenesis.

## 2. Results

### 2.1. Increased Number and Proliferation of CD34^+^ Cells in SOD1^G93A^ Rat Spinal Cord during Paralysis Progression

**Figure 1:**
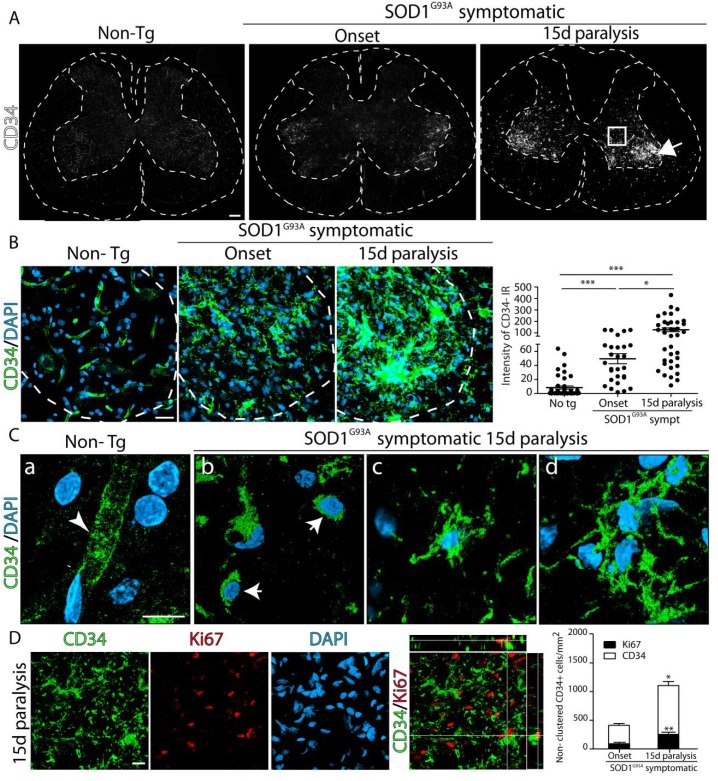
CD34^+^ immunoreactivity in the degenerating spinal cord during the course of paralysis in SOD1^G93A^ rats. Representative confocal microscopy images showing the expression of CD34 in the spinal cord of Non-transgenic (Non-Tg), SOD1^G93A^-onset, and SOD1^G93A^-15d paralysis rats. (**A**) Confocal tile reconstruction of the spinal cord showing increasing CD34 expression (white) in the ventral horn. The arrow and square indicate CD34 distribution in clustered and non-clustered cells, respectively. (**B**) Representative confocal images of CD34 expression (green) showing the detailed distribution of CD34^+^ cells in the ventral horn. Note CD34 staining in Non-Tg spinal cord was restricted to blood vessels while an increased immunoreactivity and different distribution was noted in SOD1^G93A^ rats. Dotted lines show the limit between grey and white matter in the lumbar cord. The graph to the right shows the quantitative analysis of CD34 immunoreactivity among groups. Quantitative data are expressed as mean ± SEM; data were analyzed by Kruskal–Wallis followed by Dunn’s multiple comparison test, * *p* < 0.05, *** *p* < 0.001 was considered statistically significant (**C**) Representative confocal images showing the different CD34^+^ cell phenotypes present in non-clustered regions. **a)** Blood vessels in Non-Tg animals. **b)** Two round cells. **c)** Ramified cell. **d)** Small cluster of three cells. (**D**) Confocal images showing proliferating CD34^+^ cells in non-clustered regions stained with Ki67. Orthogonal view shows Ki67^+^ nuclei on the CD34^+^ cell. The graph to the right shows the quantitative analysis of non-vascular CD34^+^ and CD34^+^/Ki67^+^ cells in non-clustered regions. Quantitative data are expressed as mean ± SEM; data were analyzed by Mann-Whitney test, * *p* < 0.05, ** *p* < 0.01 was considered statistically significant. *n* = 4 animals/condition. Scale bars: 100 μm (**A**), 25 μm (**B**), and 10 μm (**C,D**).

First, we examined the expression of CD34 in the ventral horn of the lumbar cord at paralysis onset and 15d of paralysis progression in SOD1^G93A^ rats as compared with non-transgenic rats, as shown in Figure 1A. Immunohistochemistry analysis revealed a progressive increase in CD34 expression in SOD1^G93A^ restricted to the ventral horn of the spinal cord, as shown in Figure 1A. CD34 immunoreactivity was significantly increased by 6-fold and 14-fold at paralysis onset and 15d of paralysis progression, respectively, when compared to non-transgenic rats, as shown in Figure 1B.

In control non-transgenic rats, CD34 immunoreactivity of the lumbar spinal cord was restricted to capillaries, as shown in Figure 1B,C. In symptomatic SOD1^G93A^ rats, CD34 immunoreactivity displayed two morphological patterns: (i) clusters of CD34^+^ cells containing small, round cells packed together, as shown in Figure 1B, and (ii) non-clustered, isolated CD34^+^ cells displaying rounded or ramified morphology, as shown in Figure 1C. Quantitative analysis of non-clustered CD34^+^ cells in the ventral horn showed a significant number of cells at paralysis onset, increasing by 3-fold at advanced paralysis, as shown in Figure 1D. About 15% of non-clustered CD34^+^ cells also displayed nuclear staining for the proliferation marker Ki67 at disease onset and advanced paralysis, suggesting a rapid expansion, as shown in Figure 1D.

### 2.2. CD34^+^ Cells Co-Express Myeloid and Microglia Markers

**Figure 2:**
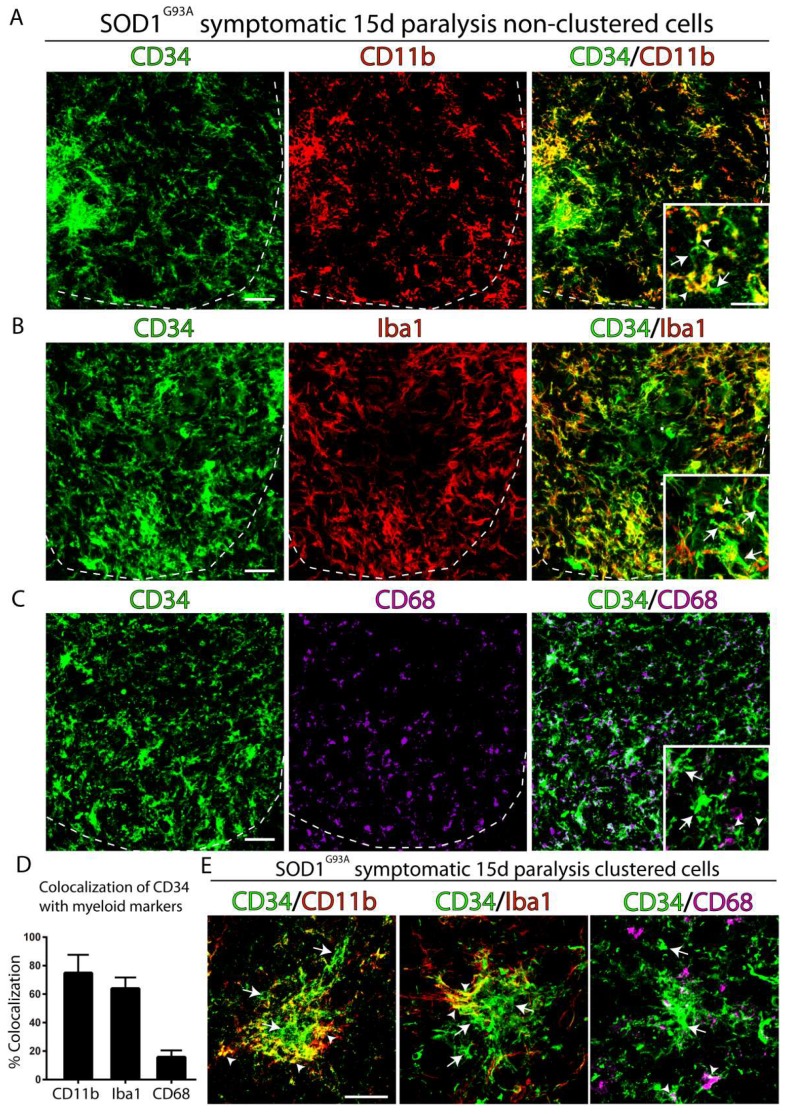
Co-expression of microglia markers and CD34. Representative confocal immunostaining of the ventral horn of symptomatic SOD1^G93A^ rat spinal cord showing the co-localization of myeloid/microglia markers CD11b (red, **A**), Iba1 (red, **B**), and CD68 (magenta, **C**). Insets show cell morphology and co-localization with CD34 at higher magnification. White arrows indicate CD34+ cells. White arrowheads indicate co-localization of CD34 with CD11b, Iba1, and CD68. Dotted lines show the limit between grey and white matter in the lumbar cord. (**D**) Confocal quantitative analysis of co-localization for CD34 and CD11b, Iba1, or CD68 in the ventral horn of symptomatic SOD1^G93A^ rat spinal cord. (**E**) Confocal analysis of the co-expression of CD34 and microglia markers in cell clusters observed in the degenerating spinal cord. Arrows indicate CD34^+^ cells in the cluster. Arrowheads indicate co-localization of CD34 with myeloid markers in the periphery of clusters. *n* = 4 animals/condition. Scale bars: 25 μm and 15 μm in insets.

Figure 2A shows that almost 80% of CD34^+^ cells in the ventral horn expressed the myeloid marker CD11b, while only 60% and 15% of cells expressed the microglia markers Iba1 or CD68, respectively, as shown in Figure 2B,C. In comparison, cells organized in large clusters mostly displayed staining for CD34 in the center and co-expressed CD11b or Iba1 in the periphery, as shown in Figure 2D, suggesting a center–periphery differentiation process.

### 2.3. CD34^+^ Cells Progressively Invade Damaged Motor Neurons Accumulating Misfolded SOD1

**Figure 3:**
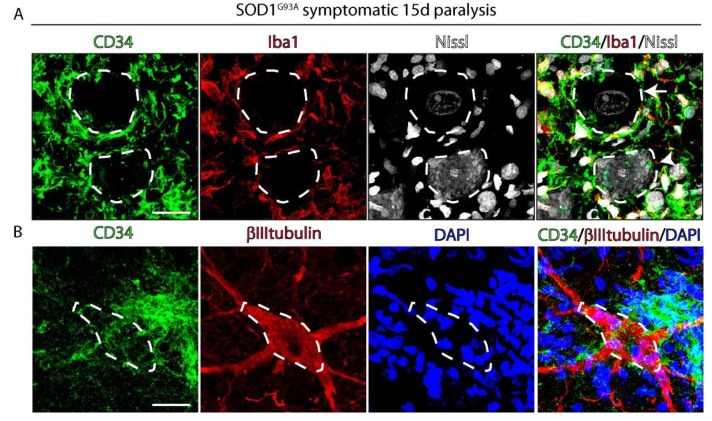
Spatial interaction of CD34^+^ cells with spinal motor neurons in symptomatic SOD1^G93A^ rats. Confocal microphotograph analyzing the association of CD34^+^ cells with motor neurons (dotted white lines) stained with Nissl (**A**) and βIII-tubulin (**B**). Note that CD34^+^/Iba1^+^ cells are adjacent to spinal motor neurons. Scale bars: 25 μm.

Figure 3 shows the early association between CD34^+^ cells and ventral horn motor neurons identified by Nissl or βIII-tubulin staining in SOD1^G93A^ rats. In non-transgenic rats, CD34 staining is restricted to blood vessels, while already in SOD1^G93A^ symptomatic onset rats CD34^+^ cells begin to surround motor neurons, as shown in Supplementary Figure S1. Typically, CD34^+^ cells locate adjacent to damaged motor neuron cell bodies and proximal neurites, which could suggest a progressive pathogenic process for individual degenerating motor neurons.

**Figure 4:**
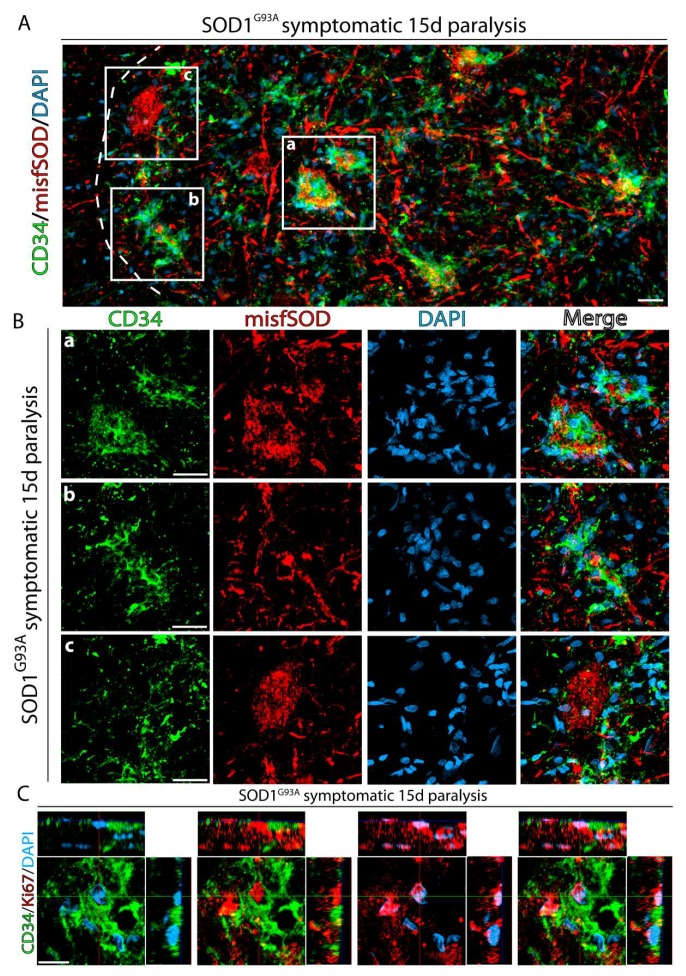
CD34^+^ cells accumulate adjacent to motor neurons expressing misfolded SOD1. (**A**) Confocal tile reconstruction of the ventral horn showing CD34^+^ cell clusters adjacent to motor neurons expressing misfolded SOD1. CD34^+^ cells completely surround and attach to damaged motor neurons expressing misfolded forms of SOD1 and form compact clusters of cells, both around motor neuron somas (**a**) and processes (**b**). Other motor neurons accumulating misfolded SOD1 have less CD34^+^ cells invading them (**c**). (**B**) Higher magnification analysis showing the clustering of CD34^+^ cells around motor neuron somas and processes. (**C**) Orthogonal view of the staining of proliferating CD34^+^ cell clusters expressing nuclear Ki67. Arrows indicate Ki67^+^ nuclei in a CD34^+^ cell cluster. Scale bars: 25 μm (**A,B**) and 10 μm (**C**).

As paralysis progressed, large motor neurons showed a tendency to lose Nissl staining and develop immunoreactivity for misfolded SOD1, indicative of dismantled endoplasmic reticulum and accumulation of misfolded proteins. Interestingly, motor neurons accumulating misfolded SOD1 were surrounded by a high number of proliferating CD34^+^ cells, as shown in Figure 4A,B, suggesting specific chemoattraction. However, as shown in Figure 4Bc, some motor neuron accumulating misfolded SOD1 may have low numbers of CD34^+^ surrounding cells. Also, these CD34^+^ cells displayed nuclear staining for Ki67, as shown in Figure 4C, suggesting a site-specific expansion of CD34^+^ cells in the surroundings of degenerating motor neurons.

### 2.4. Identification of CD34^+^ Cells in the Spinal Cord from Amyotrophic Lateral Sclerosis (ALS) Autopsied Subjects

Next, we tested the hypothesis that CD34^+^ cells also accumulate in autopsied spinal cord from ALS subjects. Supplementary Table S1 shows the characteristics of five ALS and three control donors analyzed as well as the time of postmortem tissue processing. At the time of ALS diagnosis, there was electrophysiological evidence of leg muscle denervation in each patient, supporting the involvement of the lumbar spinal cord.

**Figure 5:**
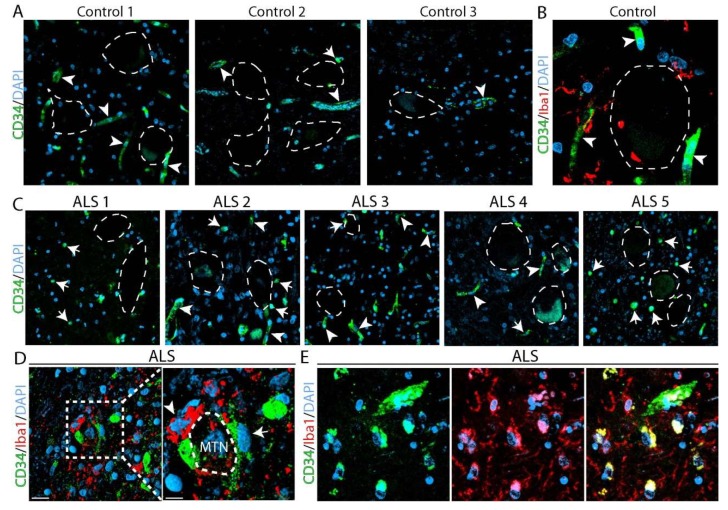
Identification of CD34^+^ cells in autopsied spinal cords from subjects with sporadic amyotrophic lateral sclerosis (ALS). Representative confocal microphotograph showing the occurrence of non-vascular CD34^+^ cells in sporadic ALS and control donors. (**A–C**) CD34^+^ cells (green) in controls and ALS subjects, respectively. Dotted lines delimitate the soma of spinal motor neurons. Note that in control subjects CD34^+^ cells were associated to blood vessels (arrowheads) with none or few rounded CD34^+^ cells located adjacent to motor neurons. In comparison, in ALS donors numerous CD34^+^ cells with round morphology were located in the proximity to apparent motor neuron cell bodies (arrows). (**D**) Confocal image showing the coexistence of Iba1^+^ cells (red) that surround an apparent motor neuron cell body (dotted lines in right panel) with CD34^+^ cells in ALS specimens. Note non-vascular CD34^+^ cells adjacent to motor neurons in ALS specimens but not in controls. (**E**) Co-expression of CD34 with Iba1 in myeloid cells at the ventral horn of the spinal cord of ALS patients. Scale bars: 25 μm (**A**), 10 μm (**B**), 50 μm (**C**), 10 μm (**D**), 20 μm (**E**).

The histological analysis of lumbar spinal cord sections showed a systematic increase in CD34^+^ cells in ALS subjects, respect to controls, where CD34^+^ immunostaining was restricted to capillaries close to motor neurons, as shown in Figure 5A,B. ALS specimens showed a decreased density of CD34^+^ immunoreactivity in capillaries. Notably, a significant number of non-vascular CD34^+^ cells with round morphology were also identified in the spinal cord of ALS subjects but not in controls, with frequent CD34^+^ cells being localized in the proximity of apparent motor neuron cell bodies morphologically identified by typical shape, size, and localization in the ventral horn as described in other reports, and as shown in Figure 5C. CD34^+^ cells in ALS subjects were not grouped in clusters and only a subpopulation of CD34^+^ cells co-expressed Iba1, as shown in Figure 5D,E.

### 2.5. Non-Adherent CD34^+^ Cells Isolated from SOD1^G93A^ Symptomatic Spinal Cord Give Rise to Microglia

**Figure 6:**
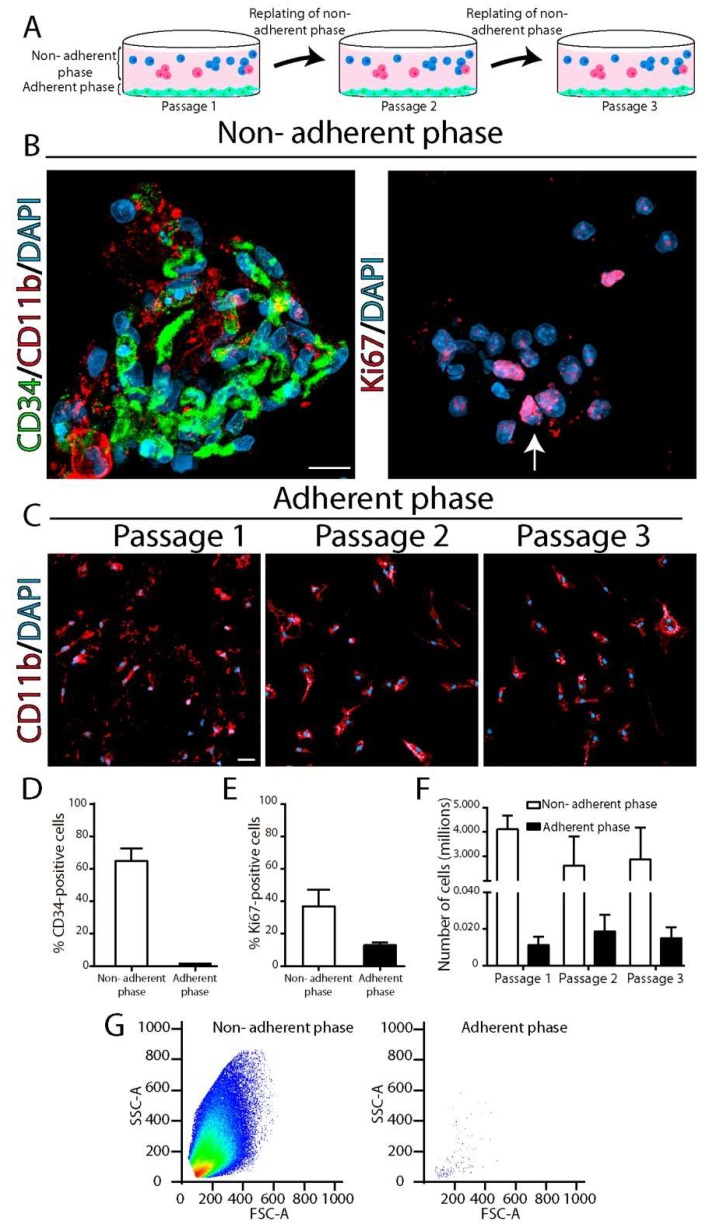
Characterization of CD34^+^ cells in primary spinal cord cultures from symptomatic SOD1^G93A^ rats. Primary cultures were prepared from symptomatic rat’s (*n* = 6) spinal cords and cells from the adherent and non-adherent phases were characterized. (**A**) Scheme showing the method used for analyzing the CD34^+^ and microglia cells (green attached cells) found in non-adherent cells (blue and red cells represent the heterogeneity of cultured non-adherent cells) after successive passages. (**B**) Cytological analysis of cell clusters found in the non-adherent phase showing CD34^+^ (green) and CD11^+^ (red, in left panel) cells as well as active proliferation as denoted by Ki67 nuclei staining (red, in right panel). (**C**) Immunostaining of adherent cells in successive passages showing sustained number of CD11b^+^ cells (red). (**D**) Quantitative analysis showing CD34^+^ cells only in the non-adherent phase. (**E**) Quantitative analysis of Ki67^+^ cells in the non-adherent and adherent phase. (**F**) Quantitative analysis showing that cells accumulate in great number in the non-adherent phase and generate adherent CD11b^+^ microglia in successive passages. (**G**) Representative plots of flow cytometry analysis showing the density of cells in both non-adherent and adherent phases. Scale bars: 10 μm (**B**) and 25 μm (**C**).

To determine the behavior of CD34^+^ cells, we isolated and cultured cells from symptomatic SOD1^G93A^ rat spinal cord. As previously reported [9], such cultures yield numerous adherent phagocytic microglia labeled with CD11b, CD68, and Iba1, as compared to only a few cells in non-transgenic controls. In the current study, CD34+ cells were not found in the adherent phase but accumulated in great number in the non-adherent phase, as shown in Figure 6A–D.

Cytological analysis of the non-adherent phase denoted that CD34^+^ cells organized in clusters of 10–20 cells, with CD34^+^ cores showing peripheral expression of CD11b, as shown in Figure 6B, suggesting a gradient of center-to-periphery differentiation. Approximately 35% of non-adherent cells displayed nuclear labeling with Ki67, indicative of active proliferation, as shown in Figure 6E.

When non-adherent cells from symptomatic SOD1^G93A^ rat spinal cords were subsequently passaged to another culture dish, numerous adherent and fully differentiated microglia were obtained in the adherent phase, as shown in Figure 6C,F,G, suggesting their ability to further differentiate as a conventional vacuolated microglia which firmly attach to the plate.

## 3. Discussion

The present study characterizes a subpopulation of CD34^+^ cells that accumulate in the ventral horn of the ALS spinal cord during the symptomatic phase of the disease. This cell type has not been previously reported in ALS. Intriguingly, these cells seem to be attracted to damaged motor neurons in the spinal cord of SOD1^G93A^ rats and in autopsied spinal cords of ALS subjects. Evidence indicates that CD34^+^ cells behave as not fully differentiated proliferating microglia, most cells expressing the broad myeloid marker CD11b. In primary cell cultures of SOD1^G93A^ symptomatic spinal cord, CD34^+^ cells are non-adherent and give rise to fully differentiated microglia, a behavior previously observed for microglia non-adherent precursor cells from the bone marrow [24]. Moreover, CD34^+^ cells exhibit an enormous proliferative capacity in SOD1^G93A^ rats forming clusters of many cells, suggesting a multi-clonal expansion process. Our findings in SOD1^G93A^rats and sporadic ALS patients are in accordance with the analysis of a public database released by a spatial transcriptome study in SOD1^G93A^ mice (https://als-st.nygenome.org/) [25], which shows a significant increase in CD34 transcripts in the lumbar spinal cord of mice over the course of the disease. Understanding the mechanism of controlling CD34^+^ precursors in ALS may have translational relevance in motor neuron diseases.

While previous reports have described the pathological features of reactive microglia and neuroinflammation in the spinal cords of ALS patients [6,26,27], this is the first observation of the occurrence of CD34^+^ cells in sporadic ALS. The finding of CD34^+^ cells surrounding motor neurons was intriguing and clearly contrasted with control subjects, where CD34 was almost exclusively expressed in capillaries. However, the number of CD34^+^ cells among sporadic ALS subjects was much lower than in the SOD1^G93A^ rats and failed to form clusters, suggesting a lower rate of proliferation. Such differences might be related to the fact that the course of the disease in rat models develops in only few weeks as compared with months to years in subjects with ALS. Also, autopsied samples from terminally ill ALS patients might not be representative of active disease and motor neuron loss. Thus, CD34^+^ cells appear to be a novel and relevant cell type involved in the ALS cellular microenvironment. It would be interesting to assess whether CD34 transcripts or CD34 protein leak into the cerebrospinal fluid of ALS patients as potential biomarkers.

The present study has not addressed the question of whether CD34^+^ cells in the spinal cord of symptomatic SOD1^G93A^ rats originate from resident microglia, perivascular macrophages, or circulating hematopoietic stem cells, a subject that would require a detailed immunophenotyping analysis. In the absence of a major blood–brain barrier breakdown, microglia expansion has been attributed to proliferation and/or migration of resident microglia [28,29,30,31], which can exhibit an impressive proliferative capacity. Here, we found that most of the CD34^+^ cells co-expressed myeloid/microglial markers such as CD11b or Iba1, suggesting the vast majority of CD34^+^ cells correspond to a subpopulation of microglia. It is possible that a subset of proliferative microglia downregulate canonical myeloid markers to levels that escaped the immunodetection. In accordance, CD34 transcripts have been detected in microglia and brain endothelial cells during development [32], while the expression of CD34 proteins is upregulated following tissue injury or blood–brain barrier disruption [22,23]. Alternatively, CD34^+^ cells in ALS could originate from the influx and subsequent expansion of blood precursor cells. In support of this mechanism, previous studies have shown that bone marrow-derived myeloid progenitors are the source for microglia in different pathological conditions, such as ischemia [32,33], Experimental autoimmune encephalomyelitis [34], and CNS axonal damage [22,35]. In ALS models, blood myeloid cells can penetrate into the spinal cord and induce pathology [36] and CD34^+^ human bone marrow cells or CD34+ blood cord cells can engraft into spinal cord capillaries and parenchyma [37,38,39,40]. Thus, it will be important to determine the origin of CD34+ microglia and the mechanism controlling their proliferation.

The remarkable cluster proliferation of CD34^+^ cells adjacent to damaged motor neurons accumulating misfolded SOD1 is intriguing and has not been previously reported in ALS. Misfolded SOD1 is considered a pathological hallmark of ALS-affected motor neurons [41,42], not only in individuals carrying SOD1 mutations but also in sporadic ALS [43]. Here, we show evidence that accumulation of misfolded SOD1 in motor neurons may stimulate the focal proliferation and clustering of CD34^+^ cells. This effect may be partially explained by the ability of misfolded SOD1 to act as a damage-associated molecular pattern activating TLR receptors [44,45]. SOD1^G93A^ mice exhibit an improved hematopoiesis compared to mice expressing wild type SOD1, suggesting a specific effect of mutant SOD1 on hematopoietic progenitors [46]. In addition, other factors produced by motor neurons upon damage may potentially induce myeloid and microglial cell attraction and proliferation, including CSF1, MCP1, and ATP, among others [47,48,49,50,51]. It remains unknown whether damaged motor neurons in ALS express stromal cell-derived factor-1α (SDF-1α), which is a potent chemokine and chemoattractant interacting with CD34 and CXCR4 in hematopoietic cells [19,52,53].

In cell culture, blood myeloid precursors are non-adherent, and adherence to substrate is characteristic of differentiation into macrophages [54,55]. Here, we provide direct evidence that, in contrast to differentiated microglia which attach to the dish, CD34^+^ cells behave as immature myeloid cells remaining in the non-adherent phase, as previously described [24]. It remains to be elucidated whether adherent fully differentiated microglia originated from a subpopulation of non-adherent CD11b^+^ cells. In addition, cultured CD34^+^ cells proliferated, formed clusters, and displayed the potential to differentiate into microglia after successive passages, as described for progenitor cells [24]. Further studies are needed to determine the stemness potential of CD34^+^ cells in the spinal cord.

In conclusion, the present study identifies CD34^+^ cells abnormally emerging in sporadic human ALS and SOD1^G93A^ rat spinal cords. Our findings broaden the myeloid/microglia phenotypic diversity in motor neuron disease. The accumulation of CD34^+^ microglia precursors around degenerating motor neurons harboring misfolded SOD1 deserves deeper mechanistic studies. CD34^+^ cells may represent a potential cell target for therapeutic development in ALS and other neurodegenerative diseases.

## 4. Materials and Methods

### 4.1. Animals

Male SOD1^G93A^ progeny, purchased from Taconic bioscience (NTac:SD-Tg(SOD1^G93A^)L26H), were used for further breeding to maintain the line [11]. Rats were housed in a centralized animal facility with a 12 h light-dark cycle with ad libitum access to food and water. Perfusion with fixative was performed under 90% ketamine/10% xylazine anesthesia and all efforts were made to minimize animal suffering, discomfort, or stress. All procedures using laboratory animals were performed in accordance with the national and international guidelines and were approved by the Institutional Animal Committee for animal experimentation. This study was carried out in strict accordance with the Institut Pasteur de Montevideo ethical committee’s requirements (CEUA Approved protocol: #005-17 to Dr. Luis Barbeito on 2^nd^ June 2017) and the national law (N° 18.611) for animal experimentation that follows the Guide for the Care and Use of Laboratory Animals of the National Institutes of Health (USA).

### 4.2. Experimental Conditions

At least 4 male rats were analyzed for the experimental condition. Non-transgenic (Non-Tg) rats were 160–180 days old, transgenic SOD1^G93A^ rats developing hind limb paralysis were differentiated at the stage of disease onset (180–190 days old), and advanced paralysis (195–210 days old) defined as 15 days after disease onset.

### 4.3. Determination of Disease Onset and End-Stage

As described previously [56], all rats were weighed and evaluated for motor activity daily. Disease onset was determined for each animal when pronounced muscle atrophy was accompanied by an abnormal gait, typically expressed as subtle limping or dragging of one hind limb. When necessary, end-stage was defined by a lack of righting reflexes or the inability to reach food and water.

### 4.4. Human Tissue Collection

The collection of post-mortem human ALS and control samples was approved by The University of Alabama at Birmingham (UAB) Institutional Review Board (Approved IRB Protocol: X091222037 to Dr. Peter H. King). All ALS patients were cared for at UAB and so detailed clinical records were available. Control samples were age-matched and were harvested from patients who expired from non-neurological causes. The average collection time after death was less than 10 h. All tissues were collected by Peter H. King and Uing Si.

### 4.5. Human Spinal Cord Immunohistochemistry

In this study, 10 μm spinal cord paraffin sections were sliced using a microtome. Following deparaffinization, slices were blocked and permeabilized in BSA 5%/Triton X-100 0.5% for 2 h at room temperature. Primary antibodies were incubated in BSA 1%/Triton X-100 0.5% at 4 °C overnight. After washing, secondary antibodies were incubated for 3 h at room temperature. After PBS washing, Mowiol medium (Sigma, St. Louis, MO, USA) was used for mounting. Only ventral lumbar spinal cord sections were analyzed. Motor neuron somas were identified in the ventral spinal cord by typical morphology and nuclei. Saturated DAPI staining was used to better differentiate motor neuron somas as shown in Supplementary Figure S2.

### 4.6. Immunohistochemistry of Spinal Cord

Four percent paraformaldehyde fixed spinal cords were cryopreserved in 30% sucrose (Sigma, St. Louis, MO, USA) at 4 °C. After 72 h, tissue was embedded in Tissue-Tek (Sakura), sectioned (longitudinal) using a cryostat, and collected on gelatin-coated slides. Then, 20 μm sections were blocked for 2 h at room temperature in 5% BSA, 0.3% Triton X-100 in PBS, incubated with primary antibodies overnight at 4 °C in BSA 1%/Triton X-100 0.3%. After washing, secondary antibodies were incubated during 2 h at room temperature in BSA 1%/Triton X-100 0.3%. To determine primary antibodies’ specificity, immunohistochemistry was carried out in the absence of primary antibodies. Non-significant immunofluorescence was detected with secondary antibodies incubation. DPX mounting medium (Sigma, St. Louis, MO, USA) was used for mounting. ImageJ software was used for analysis. For CD34 expression, cell density analysis was measured in the ventral horn of spinal cord images in at least 20 sections per spinal cord per animal (*n* = 4), as shown in Figure 1.

### 4.7. Co-Expression Analysis of CD34 and Myeloid Markers

The co-expression of CD34 with microglia markers Iba1, CD68, and CD11b was carried out using the maximum-intensity projections of images acquired from the ventral horn of spinal cord in at least 20 sections per spinal cord per animal among conditions (*n* = 4). The overlapped areas between CD34 and microglia markers were measured as previously described [57]. The number of Ki67^+^ cells co-expressing CD34 was determined by assessment on confocal 63× microphotographs of clearly identified CD34^+^ single cells in non-clustered CD34^+^ areas in at least 20 sections per spinal cord per animal among conditions (*n* = 4).

### 4.8. CD34^+^ Cell Cultures from Symptomatic SOD1^G93A^ Rats

CD34^+^ cells were obtained from a primary culture adult spinal cord of symptomatic SOD1^G93A^ rats according to the procedures described by Trias et al. (2013) [9] with minor modifications. Briefly, animals were euthanized by administering an overdose of ketamine/xylazine, and the spinal cord was dissected on ice. After the meninges were removed, the spinal cord was chopped finely and dissociated with 0.25% trypsin in a calcium-free buffer for 5 min at 37 °C. Trypsin treatment was stopped by adding DMEM/10% (vol/vol) FBS in the presence of 50 μg/mL DNaseI and mechanical disaggregation by repeated pipetting. The resulting extract was passed through an 80 μm mesh to eliminate tissue debris and was then spun. The pellet was resuspended in culture medium [DMEM/10% (vol/vol) FBS, HEPES (3.6 g/L), penicillin (100 IU/mL), and streptomycin (100 μg/mL)] and was then plated in glass-bottom p35 culture dishes for confocal microscopy or 25 cm^2^ tissue culture flasks for flow cytometry analysis. Culture medium was replaced every 48 h. As described in Figure 6, the non-adherent phase of the culture was re-plated in new p35 dishes every 48 h. Before plating every passage, adherent and non-adherent cells were quantified using a Neubauer hemocytometer. Non-adherent and adherent cells were characterized by immunocytochemistry as described below. To study the number of cells in the non-adherent and adherent phase of the primary cell culture from the adult spinal cord of symptomatic SOD1^G93A^ rats, after 2 days in vitro (DIV), both phases were analyzed by flow cytometry. Trypsin-EDTA, 0.05% (Thermo Fisher Scientific, Waltham, MA, USA) was used to remove adherent cells from the culture surface. Cells were analyzed using FlowJo software on an Attune NxT Flow Cytometer (Thermo Fisher Scientific, Waltham, MA, USA).

### 4.9. Immunocytochemical Staining of Cultured Cells

Cultured cells were fixed with 4% PFA for 20 min at 4 °C and then were washed three times with 10 mM PBS (pH 7.4). Cells were permeabilized using 0.3% Triton X-100 for 20 min. Nonspecific binding was blocked by incubating fixed cells with 5% BSA in PBS for 1 h at room temperature. Corresponding primary antibodies (see below) were diluted in blocking solution and incubated 3 h at room temperature. After washing, cells were incubated with secondary antibodies in blocking solution for 1 h at room temperature. DAPI was used for nuclei staining. At least 10 fields per plate were acquired in a confocal microscope for quantitative analysis using ImageJ software.

### 4.10. Antibodies Used

Primary antibodies used were: 1:200 rabbit monoclonal anti-CD34 (abcam, Cambridge, UK), 1:200 mouse monoclonal anti-CD34 (Thermo Fisher Scientific, Waltham, MA, USA), 1:300 mouse monoclonal anti-Iba1 (Millipore, Burlington, MA, USA), 1:200 mouse monoclonal CD68 (abcam, Cambridge, UK), 1:250 rat polyclonal anti-CD11b (BD Biosciences, Franklin Lakes, NJ, USA), 1:200 rabbit polyclonal anti-Ki67 (abcam, Cambridge, UK), 1:400 mouse monoclonal anti-GFAP (Sigma, St. Louis, MO, USA), 1:300 mouse monoclonal anti-S100β (Sigma, St. Louis, MO, USA), 1:300 mouse monoclonal anti-misfoldedSOD1 (MediMabs, Montreal, Northern Province, Canada), 1:400 mouse monoclonal anti-βIII-Tubulin (Millipore, Burlington, MA, USA). Secondary antibodies used were: 1:500 goat anti-rabbit-AlexaFluor546 or AlexaFluor633 (Thermo Fisher Scientific, Waltham, MA, USA), 1:500 goat anti-mouse-AlexaFluor488, AlexaFluor546, or AlexaFluor633 (Thermo Fisher Scientific, Waltham, MA, USA), 1:500 AlexaFluor633 (Thermo Fisher Scientific, Waltham, MA, USA).

### 4.11. Fluorescence Imaging

Fluorescence imaging was performed with a laser scanning Zeiss LSM 800 or LSM 880 confocal microscope with either a 25× (1.2 numerical aperture) objective or 63× (1.3 numerical aperture) oil-immersion objective using Zeiss Zen Black/Blue software. Maximum intensity projections of optical sections were created with Zeiss Zen software (Carl Zeiss Microscopy GmbH, Jena, Germany).

### 4.12. Statistics Analysis

Quantitative data were expressed as mean ± SEM. Two-tailed Mann–Whitney test or Kruskal–Wallis followed by Dunn’s multiple comparison test were used for statistical analysis, with *p* < 0.05 considered significant. GraphPad Prism 7.03 software (GraphPad Software, San Diego, CA, USA) was used for statistical analyses.

## Supplementary Materials

Supplementary materials can be found at https://www.mdpi.com/1422-0067/20/16/3880/s1. Table S1, Clinical characteristics of ALS and control subjects included in the study; Figure S1, Representative confocal microphotographs showing the association between CD34 and Nissl^+^ motor neurons. Note that in Non-Tg animals, CD34 was restricted to blood vessels, while in the SOD1G93A symptomatic onset, CD34 is expressed in cells that start to surround motor neurons. Figure S2, Representative confocal microphotographs showing motor neuron identification in the ventral horn of the lumbar spinal cord in sections stained with DAPI.
