# Supplementary material for: CD34 Identifies a Subset of Proliferating Microglial Cells Associated with Degenerating Motor Neurons in ALS
Source: Int J Mol Sci. 2019 Aug 9;20(16):3880. doi: 10.3390/ijms20163880 (PMC6720880; doi:10.3390/ijms20163880)
Supplement: Supplementary file 1 [file ijms-20-03880-s001.pdf]

International Journal of
Molecular Sciences

MDPI

Supplementary Table S1

Table S1. characteristics of ALS and control subjects included in the study.

Subject	Age (years)	Gender	Survival (Months)	Post-mortem Tissue Processing (hours)	EMG Denervation	Disease Onset
ALS #1	63	M	44	7.0	Active and chronic	Leg
ALS #2	69	F	50	3.0	Active	Leg
ALS #3	64	M	35	6.5	Chronic	Leg
ALS #4	59	F	26	13.0	Active	Arm
ALS #5	75	M	55	4.3	Active and chronic	Bulbar
Control #1	61	M		10.0		
Control #2	68	M		19		
Control #3	59	M		9.5		

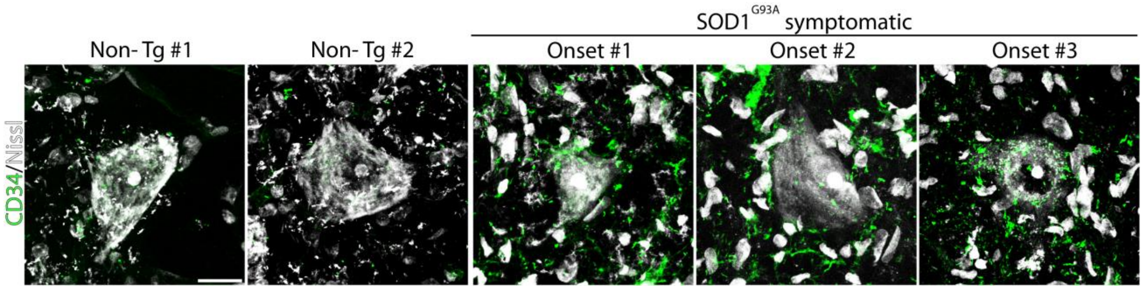

Figure S1. Representative confocal microphotographs showing the association between CD34 and Nissl+ motor neurons. Note that in Non-Tg animals, CD34 was restricted to blood vessels, while in the SOD1^{G93A} symptomatic onset, CD34 is expressed in cells that start to surround motor neurons.

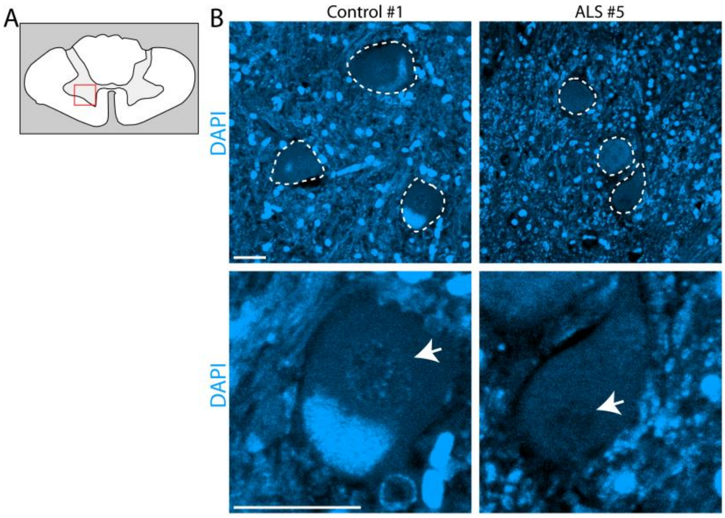

Figure S2. Representative confocal microphotographs showing motor neuron identification in the ventral horn of the lumbar spinal cord in sections stained with DAPI. (**A**) Scheme showing the human lumbar spinal cord region analyzed. (**B**) DAPI staining of one representative control and one ALS

case, showing motor neuron somas, with low and high magnification in upper and lower panels, respectively. Dotted lines indicate motor neuron cell bodies. Arrows indicate typical motor neuron nuclei. Scale bars: 50 μm.
